# Smokers’ interest in a lung cancer screening programme: a national survey in England

**DOI:** 10.1186/s12885-018-4430-6

**Published:** 2018-05-02

**Authors:** Samantha L. Quaife, Charlotte Vrinten, Mamta Ruparel, Samuel M. Janes, Rebecca J. Beeken, Jo Waller, Andy McEwen

**Affiliations:** 1 Department of Behavioural Science and Health, University College London, Gower Street, London, WC1E 6BT UK; 2 Lungs for Living Research Centre, UCL Respiratory, Division of Medicine, Rayne Building, University College London, 5 University Street, London, WC1E 6JF UK; 3 Leeds Institute of Health Sciences, University of Leeds, Worsley Building, Clarendon Way, Leeds, LS2 9NL UK

**Keywords:** Lung cancer screening, Early detection, Screening uptake, Smoking, Attitudes, Behavioural science

## Abstract

**Background:**

Following the recommendation of lung cancer screening in the US, screening committees in several European countries are reviewing the evidence for implementing national programmes. However, inadequate participation from high-risk groups poses a potential barrier to its effectiveness. The present study examined interest in a national lung cancer screening programme and modifiable attitudinal factors that may affect participation by smokers.

**Methods:**

A population-based survey of English adults (*n* = 1464; aged 50–70 years) investigated screening intentions in different invitation scenarios, beliefs about lung cancer, early detection and treatment, worry about lung cancer risk, and stigma. Data on smoking status and perceived chances of quitting were also collected, but eligibility for lung screening in the event of a national programme was unknown.

**Results:**

Intentions to be screened were high in all three invitation scenarios for both current (≥ 89%) and former (≥ 94%) smokers. However, smokers were less likely to agree that early-stage survival is good (43% vs. 53%; OR: 0.64, 0.46–0.88) or be willing to have surgery for an early stage, screen-detected cancer (84% vs. 94%; OR: 0.38, 0.21–0.68), compared with former smokers. Willingness to have surgery was positively associated with screening intentions; with absolute differences of 25% and 29%. Worry about lung cancer risk was also most common among smokers (48%), and one fifth of respondents thought screening smokers was a waste of NHS money.

**Conclusions:**

A national lung cancer screening programme would be well-received in principle. To improve smokers’ participation, care should be taken to communicate the survival benefits of early-stage diagnosis, address concerns about surgery, and minimise anxiety and stigma related to lung cancer risk.

## Background

Lung cancer is the leading cause of cancer mortality worldwide and typically has a bleak prognosis [1]; partly because early diagnoses are infrequent [2]. Low-dose computed tomography (LDCT) screening offers a means of detecting disease early, and was shown by the US National Lung Screening Trial (NLST) to reduce the relative risk of lung cancer mortality by 20%, compared with chest X-ray, for high-risk adults screened annually over 3 years [3]. Screening is recommended by the US Preventive Services Task Force (USPSTF) for current smokers and recent ex-smokers (≤ 15 years since quitting) aged 55 to 80, who have accrued a 30 pack-year smoking history [4], and is funded by Medicare and Medicaid [5]. Implementation in the UK is under review by the National Screening Committee [6].

Crucial to the effectiveness of lung cancer screening is uptake by those at high risk. This will optimise the risk-benefit ratio as the majority (88%) of deaths prevented by the NLST were for participants scoring within the three highest risk quintiles [7]. However, enrolment into trials has been low, at less than 5% of all those invited in the target age range, and biased toward those at lower risk. Current smoking status and low socioeconomic position (SEP) have predicted lower attendance across European and US trials [8–10]; the very factors associated with increased risk [11].

Surveys carried out in the community find smokers are more likely to express negative attitudes towards screening. One US population survey (*n* = 2001) found that, compared with former smokers, fewer current smokers were willing to be screened, believed early detection can increase survival, or anticipated agreeing to surgery for a screen-detected cancer [12]. In a US survey of ethnic minority groups, concerns about survival, radiation, financial cost, and the CT scan process predicted lower screening intentions [13]. More recently, an online US survey found that high perceived risk, low fear of CT scans and confidence in their accuracy, and the belief that early detection can improve prognosis, together predicted agreement to a LDCT scan [14]. In the UK, a mixed methods study of lower SEP communities suggested that fatalism about survival, risk, and treatment, and fear of an expected diagnosis may constitute important psychosocial deterrents for smokers [15]. Studies of trial non-participants have also implicated psychological deterrents, including emotional barriers such as fear, worry and avoidance [16, 17], fatalistic views and perceptions of low benefit in older age [18], and a lack of awareness that screening is beneficial for asymptomatic individuals [18]. Added to this is the potential role of social factors such as perceived stigmatisation of smoking [15, 19].

Characteristics of the invitation could also affect screening uptake. UK screening programmes for breast, colorectal and cervical cancer organise invitations through central NHS hubs, but there is good evidence that GP endorsement [20] and pre-scheduled appointments [21] improve uptake. The acceptability of these different invitation scenarios has not been studied for lung cancer screening.

This study aimed to: i) examine how screening intentions and perceptions of early detection of lung cancer might differ by smoking status, and ii) measure interest in, and acceptability of, an NHS lung cancer screening programme offered in different invitation scenarios.

## Methods

A population-based sample of adults aged 50–70 years took part in the Attitudes, Behaviour and Cancer UK Survey (ABACUS) in April 2015. This age group was selected to represent individuals who could be eligible for, or approaching eligibility for, lung cancer screening according to the USPSTF criteria [4]. The survey was administered within the rolling Omnibus survey [22] carried out by TNS Research International, which uses home-based computer-assisted, face-to-face interviews. Sampling points in England were selected using stratified random location sampling from the 2011 Census small area statistics [23], the Postcode Address File and Government Office Regions. At each sampling location, quotas were set for age, gender, children residing in the household, and employment status.

### Measures

A brief, standardised description of lung cancer screening was provided and single-item questions were adapted from existing measures and studies, and piloted in cognitive interviews (*n* = 15), and an online survey (*n* = 391) prior to this study.

### Lung cancer screening intentions

Participants were asked to rate their intention to be screened following three hypothetical invitation scenarios presented in the same order to all participants: i) an invitation from a national NHS programme, ii) a GP recommendation, and iii) an upcoming pre-scheduled appointment next month. These items were adapted from the colorectal cancer screening literature [24]. It was made clear to participants that there is currently no national lung cancer screening programme in England. Responses were on a five-point scale for the first two items (‘yes definitely’, ‘yes probably’, ‘probably not’, ‘definitely not’, ‘not sure’) and the third item (‘very likely’, ‘likely’, ‘unlikely’, ‘very unlikely’, ‘not sure’). They were dichotomised for analysis as ‘yes definitely/probably’ vs. ‘probably/definitely not’, and ‘very likely/likely’ vs. ‘very unlikely/unlikely’. Those answering ‘not sure’ or ‘refused’ on any of the three items were excluded.

### Beliefs about lung cancer survival, early detection and screening

Two items were taken from the Awareness and Beliefs about Cancer (ABC) measure [25] concerning survival from cancer and lung cancer (‘a diagnosis of cancer/lung cancer is a death sentence’). Response options were on a four-point scale dichotomised as ‘strongly/tend to agree’ vs. ‘strongly/tend to disagree’ for analysis. Participants could answer ‘don’t know’ or ‘refused’, but these responses were excluded from analyses. Participants who had been diagnosed with cancer (*n* = 127) were not asked these questions.

Two items were adapted from Silvestri and colleagues’ US survey [12] to assess beliefs about early-stage lung cancer: ‘If lung cancer is detected early, what is the person’s chance of surviving?’ (response options on a three-point scale, dichotomised for analysis as ‘good’ vs. ‘fair/poor’) and ‘If the screening test found that you had early-stage lung cancer, would you want to have the recommended surgery?’ (responses were coded as ‘yes definitely/probably’ or ‘probably/definitely not’). ‘Not sure’, ‘don’t know’ or ‘refused’ responses were excluded.

The acceptability of a screening programme was also assessed (‘Do you think lung cancer screening is a good idea?’), as well as opposition against screening targeted at smokers as an indicator of stigma (‘Do you think that offering lung cancer screening to smokers is a waste of NHS money?’) adapted from a validated cancer stigma scale [26]. Response options were ‘yes’ or ‘no’; ‘don’t know’ or ‘refused’ responses were excluded.

### Worry about lung cancer risk

Frequency of worry about lung cancer risk was measured by asking, ‘How often do you worry about your chance of getting lung cancer?’; adapted from Lerman’s Cancer Worry Scale [27, 28]. Response options were ‘never’, ‘occasionally’, ‘sometimes’, ‘often’, ‘very often’, which were recoded as ‘never’ vs. ‘at least occasionally’ for analysis. Respondents who reported worry were asked, ‘Would a clear lung CT scan reassure you?’, to which they could respond ‘yes’ or ‘no’. ‘Don’t know’ and ‘refused’ responses were excluded from analyses.

### Smoking

Smoking status was self-reported using the following two items from the ABC measure [25]. First, ‘Do you smoke at all these days, either cigarettes (including hand-rolled ones), pipes or cigars?’ and second, ‘Have you ever regularly smoked cigarettes (including hand-rolled ones), pipes or cigars?’. Former smokers were therefore defined as individuals who had ever smoked tobacco regularly. We did not collect data on tobacco consumption or smoking duration and therefore could not determine likely screening eligibility status.

Current smokers were asked, ‘How high would you rate your chances of giving up smoking for good?’ on a scale adapted from the ‘Motivation To Stop Scale’ (MTSS) [29]. Responses were dichotomised as high (‘extremely’, ‘very high’, ‘quite high’) vs. low (‘not very high’, ‘low’ or ‘very low’) for analysis.

### Demographics

Data on age, gender, ethnicity (White/Not White), marital status (i) married/cohabiting, ii) single/divorced/separated/widowed), and highest level of education (i) no formal qualifications, ii) CSEs/O-levels/equivalent, iii) A-levels/further education/equivalent, iv) Degree or higher) were collected. Cancer experience was assessed: ‘Have any friends or family members that are close to you ever been diagnosed with cancer?’ to which participants could answer ‘yes’ or ‘no’.

### Analyses

Descriptive frequencies were run to determine absolute levels of agreement. The associations between smoking status and agreement with each cancer belief and worry item were explored using chi-square analyses, and multivariable logistic regression, adjusted for demographics and cancer experience. ‘Don’t know’ (≤ 6.9%) and ‘refused’ (≤ 4.9%) responses were excluded from the respective analyses.

Chi-square and logistic regression analyses were also carried out to test for associations between demographics, smoking status, and screening intentions. Belief items associated with smoking status were then analysed to determine their association with screening intentions. Analyses of screening intentions excluded never smokers because they would not be eligible for lung cancer screening. Analyses also excluded cases answering ‘not sure’ (≤ 2.5%) or ‘refused’ (≤ 2.3%) on any one of the three intention items (*n* = 44). Further analyses tested for demographic differences between this group and the overall sample.

Finally, exploratory chi-square and logistic regression analyses tested for associations between quit confidence and the beliefs, lung cancer worry and screening intention variables among current smokers only. As multiple testing increases the type one error rate, a stringent significance threshold was set for the interpretation of all analyses (*p* < .01).

## Results

### Sample characteristics

In total, 1464 participants completed the survey. Participants were excluded if they did not report their smoking status (*n* = 13) or had been diagnosed with lung cancer (*n* = 6). The average age of the final sample was 60 years and there was good representation of different SEP (as indicated by education level; see Table 1). The majority were married or cohabiting (62%), and from a White ethnic background (93%). Experience of cancer through friends or family was commonly reported (70%).

**Table 1:** Characteristics of the sample by smoking status

	All (*n* = 1445)	Never smokers (*n* = 759)	Current smokers (*n* = 313)	Former smokers (*n* = 373)
Gender, % (n)				
Male	49.3 (712)	44.0 (334)^b^	55.9 (175)^b^	54.4 (203)^b^
Female	50.7 (733)	56.0 (425)	44.1 (138)	45.6 (170)
Age, mean (SD)	60.4 (6.3)	60.1 (6.3)	59.2 (6.3)	61.8 (6.1)
Marital status, % (n)				
Married/Cohabiting	61.7 (892)	65.3 (496)^b^	48.2 (151)^b^	65.7 (245)^b^
Single/Divorced/Separated/Widowed	38.3 (553)	34.7 (263)	51.8 (162)	34.3 (128)
Ethnicity, % (n)				
White	92.9 (1342)	89.3 (678)^b^	95.5 (299)^b^	97.9 (365)^b^
Not White	6.9 (100)	10.4 (79)	4.5 (14)	1.9 (7)
Refused	0.2 (3)	0.3 (2)	0.0 (0)	0.3 (1)
Education level, % (n)				
Degree	20.2 (292)	26.2 (199)^b^	10.2 (32)^b^	16.4 (61)^b^
A-levels/further/equivalent	22.6 (327)	24.0 (182)	16.9 (53)	24.7 (92)
CSEs/O-levels/equivalent	28.3 (409)	26.0 (197)	33.5 (105)	28.7 (107)
No formal qualifications	26.1 (377)	21.2 (161)	36.4 (114)	27.3 (102)
Don’t know/Refused	2.8 (40)	2.6 (20)	2.9 (9)	2.9 (11)
Cancer experience, % (n)				
Yes (friends/family)	70.2 (1014)	68.8 (522)	69.0 (216)	74.0 (276)
No	28.2 (407)	30.0 (228)	29.1 (91)	23.6 (88)
Don’t know/Refused	1.7 (35)	1.2 (9)	1.9 (6)	2.4 (9)
Quit confidence, % (n)				
Extremely high	–	–	4.2 (13)	–
Very high	–	–	10.2 (32)	–
Quite high	–	–	20.1 (63)	–
Not very high	–	–	21.7 (68)	–
Low	–	–	13.7 (43)	–
Very low	–	–	26.2 (82)	–
Don’t know/Refused	–	–	3.8 (12)	–

% totals may not be exactly 100 due to rounding

^a^χ^2^, *p* < .01, ^b^ χ^2^, *p* < .001

Twenty two per cent of participants were current smokers, 26% were former smokers and 52% reported never having smoked. Current smokers had a lower level of education, and were less likely to be married (p’s < .001). Most smokers (62%) rated their chances of stopping smoking as ‘very low’, ‘low’ or ‘not very high’. Beyond age and smoking status, participants’ likely eligibility in the event of a national lung cancer screening programme was unknown.

Compared with the main sample, current and former smokers answering ‘don’t know’ or ‘refused’ on the intention items (*n* = 44; excluded from the intention analyses) did not differ in their sociodemographic characteristics.

### Beliefs about lung cancer survival, early detection and screening

One in five respondents agreed that a cancer diagnosis is a death sentence, but this number doubled (48%) when the question concerned lung cancer (see Table 2). In relation to early-stage lung cancer, only half thought the chances of surviving were good, but 92% anticipated they would opt for surgery. Smokers were less likely to agree with these beliefs compared with former smokers (43% vs. 53%, *p* = .01; OR: 0.64, 0.46–0.88, and 84% vs. 94%, *p* < .001; OR: 0.38, 0.21–0.68 respectively).

**Table 2:** Frequencies, chi-square analyses and multivariable logistic regression models^c^, testing the associations between smoking status, beliefs and worry

	All (n = 1445)	Former smokers (reference)		Current smokers			Never smokers		
	% (n)	% (n)	OR	% (n)	OR	95% CI	% (n)	OR	95% CI
*A diagnosis of cancer is a death sentence*									
Strongly/tend to agree (vs. strongly/tend to disagree)	20.5 (257)	16.9^a^ (53)	1.00	26.8^a^ (75)	1.50	0.97–2.29	19.6^a^ (129)	1.29	0.88–1.90
*A diagnosis of lung cancer is a death sentence*									
Strongly/tend to agree (vs. strongly/tend to disagree)	47.6 (582)	46.3 (138)	1.00	53.2 (149)	1.25	0.89–1.76	45.7 (295)	1.01	0.76–1.35
*If lung cancer is detected early, what is the person’s chance of surviving?*									
Good (vs. fair/poor)	50.8 (689)	53.4 (189)	1.00	43.0 (125)	0.64	0.46–0.88	52.7 (375)	0.95	0.73–1.24
*If the screening test found that you had early-stage lung cancer, would you want to have the recommended surgery?*									
Yes definitely/probably (vs. definitely/probably not)	91.5 (1209)	93.9^b^ (321)	1.00	84.3^b^ (242)	0.38	0.21–0.68	93.4^b^ (646)	0.99	0.56–1.75
*Do you think that lung cancer screening is a good idea?*									
Yes (vs. no)	97.1 (1354)	97.8 (352)	1.00	96.7 (294)	0.67	0.25–1.77	97.0 (708)	1.10	0.46–2.63
*Do you think that offering screening to smokers is a waste of NHS money?*									
Yes (vs. no)	20.7 (281)	23.9^a^ (84)	1.00	14.2^a^ (43)	0.45	0.29–0.69	21.9^a^ (154)	0.92	0.67–1.27
*How often do you worry about your chance of getting lung cancer?*									
Very often to occasionally (vs. never)	30.6 (429)	28.7^b^ (104)	1.00	47.9^b^ (147)	2.38	1.70–3.33	24.4^b^ (178)	0.81	0.60–1.10
*Would a clear CT scan reassure you?* ^d^									
Yes (vs. no)	90.0 (385)	94.2 (97)	1.00	89.7 (130)	0.66	0.24–1.84	87.8 (158)	0.75	0.27–2.05

*OR* odds ratio, *95% CI* 95% confidence interval; n totals may not sum due to missing data

^a^χ^2^, *p* < .01, ^b^ χ^2^, *p* < .001

^c^adjusted for demographics and cancer experience

^d^asked of the subsample of participants reporting lung cancer worry (*n* = 429)

The large majority of participants (97%) thought screening a good idea, across smoking groups. Using NHS money to screen smokers was perceived as a waste of NHS money by 21%, but most commonly by former (24%) and never smokers (22%) compared with current smokers (14%; OR: 0.45, 0.29–0.69; reference group was former smokers).

While there was a trend towards smokers with a lower perceived chance of stopping smoking endorsing more negative beliefs, there were no statistically significant associations in unadjusted analyses (*n* = 301; see Table 3). In adjusted analyses, smokers who perceived their chance of quitting as low were less likely to agree that early stage survival is good compared with those rating their chance of quitting as high (37% vs. 52%, *p* = .02; OR: 0.48, 0.29–0.82).

**Table 3:** Frequencies, chi-square analyses, and multivariable logistic regression models^a^, exploring associations between smokers’ perceived chance of quitting, lung cancer beliefs and screening intentions

	High quit confidence (reference)		Low quit confidence		
	% (n)	OR	% (n)	OR	95% CI
*A diagnosis of cancer is a death sentence*					
Strongly/tend to agree (vs. strongly/tend to disagree)	25.5 (26)	1.00	28.2 (48)	1.23	0.68–2.24
*A diagnosis of lung cancer is a death sentence*					
Strongly/tend to agree (vs. strongly/tend to disagree)	50.0 (50)	1.00	53.8 (93)	1.14	0.68–1.92
*If lung cancer is detected early, what is the person’s chance of surviving?*					
Good (vs. fair/poor)	51.9 (54)	1.00	37.3 (66)	0.48	0.29–0.82
*If the screening test found that you had early-stage lung cancer, would you want to have the recommended surgery?*					
Yes definitely/probably (vs. definitely/probably not)	88.0 (88)	1.00	81.3 (143)	0.58	0.27–1.24
*Do you think that lung cancer screening is a good idea?*					
Yes (vs. no)	96.3 (103)	1.00	96.8 (180)	1.11	0.29–4.20
*Do you think that offering screening to smokers is a waste of NHS money?*					
Yes (vs. no)	10.4 (11)	1.00	16.1 (30)	1.62	0.76–3.47
*How often do you worry about your chance of getting lung cancer?*					
Very often to occasionally (vs. never)	48.6 (52)	1.00	47.6 (90)	0.92	0.56–1.51
*Would a clear CT scan reassure you?* ^b^					
Yes (vs. no)	92.2 (47)	1.00	87.6 (78)	0.48	0.13–1.84
NHS invitation					
Yes definitely/probably (vs. definitely/probably not)	93.2 (96)	1.00	86.4 (159)	0.55	0.22–1.37
GP recommendation					
Yes definitely/probably (vs. definitely/probably not)	94.3 (99)	1.00	92.0 (172)	0.82	0.29–2.29
Appointment next month					
Very likely/likely (vs. very unlikely/unlikely)	94.2 (98)	1.00	86.6 (161)	0.46	0.18–1.21

*OR* odds ratio; *95% CI* 95% confidence interval

^a^adjusted for demographics and cancer experience

^b^asked of the subsample of participants reporting lung cancer worry (*n* = 142)

### Worry about lung cancer risk

Worrying about risk of lung cancer at least occasionally was fairly common overall (31%; see Table 2). More current smokers reported this worry (48%), compared with former smokers (29%; OR: 2.38, 1.70–3.33), and fewer never smokers compared with former smokers (24% vs. 29%, *p* < .001); although the latter association was not statistically significant in adjusted analyses (OR: 0.81, 0.60–1.10). Of the participants who worried about their risk (*n* = 429), 90% thought a clear CT scan would be reassuring, with no association with smoking status.

### Lung cancer screening intentions

The large majority of current and former smokers intended to be screened for lung cancer (see Table 4). The proportion of intenders was highest if recommended by a GP (93% and 98%, for current and former smokers respectively), and was similar for the NHS (89% and 94% for current and former smokers) and upcoming appointment (89% and 94%) invitation scenarios.

**Table 4:** Frequencies, chi-square analyses, and multivariable logistic regression models^c^, exploring associations with lung cancer screening intentions among current and former smokers

	NHS invitation^d^			GP recommendation^d^			Appointment next month^e^		
	Intend % (n)	OR	95% CI	Intend % (n)	OR	95% CI	Intend % (n)	OR	95% CI
*All (n = 642)*	91.6 (588)	–	–	95.8 (615)	–	–	91.9 (590)	–	–
Gender									
Female	91.6 (261)	1.00	–	95.8 (273)	1.00	–	93.0 (265)	1.00	–
Male	91.6 (327)	1.06	0.59–1.90	95.8 (342)	1.01	0.44–2.33	91.0 (325)	0.78	0.42–1.44
Age	–	1.01	0.97–1.06	–	0.96	0.89–1.02	–	0.99	0.94–1.04
Marital status									
Married/Cohabiting	92.8 (347)	1.00	–	96.5 (361)	1.00	–	92.8 (347)	1.00	–
Single/Divorced/Widowed	89.9 (241)	0.69	0.26–1.24	94.8 (254)	0.60	0.26–1.39	90.7 (243)	0.69	0.37–1.27
Education level									
Degree	88.8 (79)	1.00		96.6 (86)	1.00	–	94.4 (84)	1.00	–
A-levels/further/equivalent	92.9 (130)	1.40	0.54–3.62	96.4 (135)	0.97	0.22–4.26	93.6 (131)	0.86	0.28–2.70
CSEs/O-levels/equivalent	92.5 (184)	1.55	0.64–3.73	96.5 (192)	1.24	0.31–5.04	91.0 (181)	0.68	0.24–1.93
No formal qualifications	91.6 (185)	1.35	0.56–3.24	95.0 (192)	1.04	0.27–4.08	92.1 (186)	0.84	0.29–2.42
Cancer experience									
Yes	92.3 (432)	1.00	–	96.4 (451)	1.00	–	93.2 (436)	1.00	–
No	89.9 (151)	0.67	0.36–1.26	94.0 (158)	0.53	0.23–1.23	88.1 (148)	0.48	0.26–0.89
Smoking status									
Former	93.7 (328)	1.00	–	98.3 (344)^a^	1.00	–	94.0 (329)	1.00	–
Current	89.0 (260)	0.67	0.36–1.24	92.8 (271)^a^	0.24	0.09–0.65	89.4 (261)	0.63	0.33–1.19
Early-stage survival									
Good	94.4 (289)	1.00	–	98.4 (301)^a^	1.00	–	94.4 (289)	1.00	–
Fair/Poor	89.4 (279)	0.46	0.24–0.87	93.9 (293)^a^	0.23	0.08–0.71	90.7 (283)	0.55	0.28–1.06
Early-stage surgery									
Yes definitely/probably	94.3 (512)^b^	1.00	–	98.7 (536)^b^	1.00	–	95.4 (518)^b^	1.00	–
Definitely/probably not	69.4 (43)^b^	0.14	0.07–0.28	74.2 (46)^b^	0.04	0.02–0.12	66.1 (41)^b^	0.09	0.04–0.20
Lung cancer worry									
Never	90.5 (364)	1.00	–	95.8 (385)	1.00	–	90.5 (364)	1.00	–
Occasionally to very often	93.7 (223)	1.68	0.88–3.20	96.2 (229)	1.44	0.60–3.46	94.5 (225)	1.80	0.91–3.57

*OR* odds ratio; *95% CI* 95% confidence interval; n totals may not sum due to missing data

^a^χ^2^, *p* < .01; ^b^ χ^2^, *p* < .001

^c^adjusted for demographics, cancer experience and smoking status

^d^predicting yes definitely/probably vs. probably/definitely not

^e^predicting very likely/likely vs. very unlikely/unlikely

Gender, age, ethnicity, level of education, marital status and cancer experience were not associated with screening intentions. Smoking status was associated with screening intentions in the GP invitation scenario only. Fewer current smokers (93%) than former (98%) smokers intended to participate following a GP recommendation (*p* < .01; OR: 0.24, 0.09–0.65). Smokers’ perceived chance of quitting smoking was not associated with their intentions to be screened.

Perceived survival from early-stage lung cancer was associated with screening intentions in the GP invitation scenario (see Table 4), with decreased odds for those thinking survival was poor or fair (94%), compared with good (98%, *p* < .01; OR: 0.23, 0.08–0.71). Anticipating not wanting surgery for a screen-detected early-stage lung cancer predicted a lower likelihood of intending to be screened in all three scenarios. Striking absolute differences in intentions were observed between the ‘decline’ and ‘pro’ surgery participants (26–29%; ORs: 0.04–0.14, *p* < .001).

Worrying about risk of lung cancer did not affect the odds of intending to be screened in any of the invitation scenarios. However, this group was predominantly comprised of individuals who worried sometimes or occasionally (85% of the current and former smokers reporting worry and included in the screening intention analyses).

There were too few cases to subdivide the frequency of worry by screening intentions for multivariate analysis. The results of unadjusted Fisher’s exact tests suggested that *infrequent worry* (i.e. occasionally or sometimes) was associated with higher screening intentions, whereas *frequent worry* (i.e. often or very often) was associated with lower intentions in the NHS invitation scenario (*p* = .01; results not reported).

## Discussion

This is the first UK population-based study of older adults to investigate interest in, and perceptions of lung cancer screening, and to explore their association with smoking status. Most respondents thought screening a good idea and the majority of current and former smokers intended to be screened. However, positive intentions were at odds with frequently fatalistic perceptions of lung cancer. Negative beliefs about early-stage survival and surgery were particularly common among smokers, and associated with a lower likelihood of intending to be screened.

Based on intentions alone, these findings suggest that a UK national screening programme would be acceptable and well-attended; perhaps especially if recommended by a GP. These findings match the interest observed among a US population-based survey [12]. However, they are contrary to the low levels of screening uptake observed in the trial context [10]. The gap between intentions and behaviour is well-documented for health precautionary behaviours [30] suggesting these intentions may not be borne out in attendance. Cognitive, psychosocial or practical factors may subsequently determine whether intentions are enacted.

Beliefs about the screened disease could be an important factor. Perceptions of survival from lung cancer were frequently negative; even when early detection was specified. This is likely to reflect the poor prognosis lung cancer currently has, due largely to its late diagnosis. A deep-rooted lay interpretation might be that outcomes are universally poor, and there is evidence that early-stage survival is underestimated [31]. Notably, smokers were the most negative. Fewer believed there is a good chance of surviving early-stage lung cancer, or that they would undergo surgery for a screen-detected cancer, mirroring the results of the US survey [12]. Smokers in this UK sample were more accepting of surgical treatment (84% vs. 56%), which could partly be due to differences in healthcare provision, as US smokers were more likely to be deterred by cost. Importantly, those holding negative beliefs about early-stage survival and surgery were less likely to intend to be screened, suggesting that negative perceptions of early detection could undermine smokers’ screening intentions.

Smokers’ greater pessimism about early-stage lung cancer may result from more negative experiences of the disease within their social networks. We adjusted for previous cancer experience, but did not assess the type. Alternatively, perhaps smokers in this relatively older age group have become increasingly fatalistic about their chances of surviving lung cancer because of their significant smoking history, such that screening and treatment are perceived as offering little promise. These feelings may be exacerbated by their tobacco dependence, especially if they feel unable to quit; important because a sizeable proportion of smokers (> 60%) rated their chances of quitting as low. While perceived chance of quitting was not associated with screening intention, this study provided preliminary evidence that lower perceptions of quitting could foster more negative perceptions of early detection for lung cancer. This hypothesis deserves further study.

Worry about risk of lung cancer was most prevalent among smokers; nearly half reported worrying at least occasionally. Overall, worry did not appear to affect the likelihood of intending to be screened among current and former smokers. However, subgroup analyses showed that *frequent worriers* actually had lower intentions to be screened, and that *infrequent worriers* had the highest intentions in the NHS invitation scenario. Although preliminary, these results are consistent with evidence for a curvilinear association [32]. Studies are needed to measure the constituent components of smokers’ worry, including frequency, and to explore their effects on screening participation.

While many respondents worried about their risk of lung cancer, most believed they would find a clear screen reassuring. However, this belief may be irrelevant if screening is expected to lead to a lung cancer diagnosis, and smokers may therefore be less likely to anticipate reassurance. Previous data have shown smokers are more likely to delay symptomatic help-seeking [33] due to worry about what the doctor might find [34]. Studies have also warned that lung cancer screening could undermine motivation to stop smoking [35]. However, with the correct communication, screening offers the opportunity to both assist smoking cessation and raise symptom awareness. Research is needed which explores the psychological responses to different screening results to identify how best to communicate lung cancer risk to assist positive behaviour change.

Lung cancer screening would be the first cancer screening programme in the UK to select patients primarily using a behavioural risk factor. In this sample, nearly one fifth thought screening smokers would be a waste of NHS money, suggesting that the stigma attached to smoking may adversely affect the acceptability of a targeted programme. This finding also warns that the exclusion of never smokers could be a contentious issue as they can also develop lung cancer, and a quarter of never smokers reported worrying about this. Opposition to their exclusion should be addressed by carefully communicating why LDCT screening is only appropriate for those at high risk.

This study benefits from a large, population-based sample, with a higher proportion of smokers (22%) than was expected for this age group [36] and good representation of different SEP groups. The measurement of screening intentions was useful for exploring interest, but limited conclusions can be made about screening behaviour, because the two are not well-correlated [30]. This may have been exacerbated by the fact that the screening offer was hypothetical and social desirability bias may have inflated results. Furthermore, asking participants about multiple invitation scenarios may have led them to alter their response for subsequent scenarios relative to the previous scenarios. This method could have resulted in different invitation preferences compared to asking independent groups about each invitation scenario; although respondents could not alter their previous responses when interviewed. Alternative individual-level measures of SEP exist but education has been shown to be a good indicator in older samples [37]. Single-item, cross-sectional measures were chosen to minimise participant burden, but may have reduced the reliability of findings. For the same reason, we did not collect information on participants’ smoking duration or history (i.e. pack years) which would have allowed us to determine their eligibility for lung cancer screening. We caution that not knowing the likely eligibility status of participants in our sample may reduce the generalisability of these findings to an eligible screening population in England, should a national screening programme be recommended.

## Conclusions

The introduction of an NHS lung cancer screening programme appears to be acceptable to older UK adults, with most current and former smokers intending to be screened (upwards of 89% and 94%, respectively) especially if recommended by their GP. Smokers’ greater pessimism about survival and treatment for early-stage cancer could help to explain their lower participation. Strategies aimed at engaging smokers with screening should focus on improving perceptions of the curability of early-stage disease and addressing concerns about surgical treatment. Communication throughout the screening process needs to be sensitively devised so that it is mindful of the existing stigma around smoking, and the anxiety smokers may have about their increased risk of lung cancer.

## Abbreviations

ABACUS: Attitudes behaviour and cancer UK survey
ABC: Awareness and beliefs about cancer measure
GP: General practitioner
LDCT: Low-dose computed tomography
MTSS: Motivation to stop scale
NHS: National health service
SEP: Socioeconomic position
USPSTF: United States preventive services task force
